# Therapeutic Alliance in Family-Based Treatment of Anorexia Nervosa: In-Person Versus Telehealth

**DOI:** 10.1002/cpp.3017

**Published:** 2024

**Authors:** Marita Cooper, Chloe Connor, Natalia Orloff, John D. Herrington, C. Alix Timko

**Affiliations:** 1 Department of Child and Adolescent Psychiatry and Behavioral Sciences, Children's Hospital of Philadelphia, Philadelphia, Pennsylvania, USA; 2 Department of Psychiatry, Perelman School of Medicine, University of Pennsylvania, Philadelphia, Pennsylvania, USA

**Keywords:** anorexia nervosa, family-based treatment, telehealth, therapeutic alliance, working alliance

## Abstract

**Objective:**

The therapeutic alliance is broadly linked with positive outcomes. However, nearly all research in this area involves in-person therapy, whereas teletherapy has grown increasing common since the COVID-19 pandemic. There is now a pressing need to establish whether the nature and importance of the therapeutic alliance is impacted by teletherapy. This study examined therapeutic alliance in families of youth with anorexia nervosa who were participating in a randomized controlled trial that transitioned from in-person to telehealth visits during the COVID-19 pandemic.

**Method:**

We analysed data from 53 adolescents and their parents (20 began in-person, 33 began with telehealth). Both parents, youth and therapist completed the Working Alliance Inventory–Short Revised after 4 weeks of treatment.

**Results:**

We found no significant differences across telehealth and in-person treatment for paternal or therapist reported data. However, both adolescents and mothers reported higher bond and goal-related alliance for in-person sessions compared to telehealth.

**Conclusions:**

Findings regarding alliance across telehealth and in-person sessions were mixed, with some preference among mothers and youth for in-person treatment. Future studies should determine whether possible adaptations can improve working alliance during family-based treatment for anorexia nervosa via telehealth.

## 1 ∣ Introduction

The COVID-19 pandemic, and related public health precautions, led to a rapid shift from in-person psychotherapy to remote telehealth sessions worldwide (Fisk, Livingstone, and Pit 2020). Telehealth options have generally been well-received by patients, who report preferring the convenience and increased accessibility of at-home therapy (Andrews et al. 2020; Couturier et al. 2022; Frayn, Fojtu, and Juarascio 2021). In contrast, many clinicians have raised concerns about the impact of telehealth on alliance/rapport building (Cowan et al. 2019; Madigan et al. 2021). These concerns may be particularly pertinent for clinicians working with youth with anorexia nervosa (AN), given the ambivalence toward treatment seen in many adolescents with AN and the ego-syntonic nature of the condition (Matheson, Bohon, and Lock 2020). Generally, strong positive therapeutic alliance is linked to favourable treatment outcomes across a broad range of psychiatric disorders (Horvath et al. 2011; Shirk, Karver, and Brown 2011), although data linking therapeutic alliance and outcome in AN are more nuanced. For example, weaker parent therapeutic alliance is linked to treatment dropout and poorer early weight gain, yet neither adolescent nor parent therapeutic alliance appears to predict end-of-treatment remission in youth with AN (Forsberg et al. 2014; Graves et al. 2017; Pereira, Lock, and Oggins 2006). Given the proliferation of telehealth since the COVID-19 pandemic, it is critical to examine how therapeutic alliance when treating youth with AN may have been impacted by this transition.

For individuals with eating disorders, data on the therapeutic alliance during the transition to telehealth have been mixed (Linardon et al. 2022). Raykos et al. (2021) found that most participants described telehealth either as comparable to or better than in-person treatment, while most participants in a study by Lewis et al. (2021) noted they would prefer to return to in-person therapy. Similarly in a study of youth undergoing family-based treatment (FBT), parents reported greater comfort completing therapy in their home, but noted feeling that technical difficulties (e.g., audio/video lag) impaired their therapeutic engagement (Couturier et al. 2022). However, studies have been predominantly qualitative, and participants have typically opted into participation in a telehealth study rather than being recruited through an in-person trial—potentially biasing recruitment toward those with highly negative or positive opinions of telehealth.

This post hoc study explored therapeutic alliance as reported by both therapist and family members (mother, father and adolescent) in a clinical trial providing FBT to youth with AN. As the trial was ongoing during the pandemic, we were able to compare alliance in families who started in-person treatment before the initial COVID-19 lockdowns to those who received telehealth for the duration. We focused on therapeutic alliance at 4 weeks after treatment initiation as this is a key clinical timepoint; adolescent weight gain by 4 weeks significantly predicts weight restoration (Martin-Wagar, Holmes, and Bhatnagar 2019). As this is among the first studies to examine therapeutic alliance differences between in-person and telehealth-driven AN treatment, we did not have specific hypotheses.

## 2 ∣ Method

### 2.1 ∣ Participants

We conducted secondary data analysis on data from a randomized controlled trial (see Timko et al. 2021) of 59 adolescents with AN and their parents between August 2019 and December 2020. Three families dropped out of the study prior to Week 4 of treatment (T2), two families did not complete T2 assessment and one family was unable to be classified as telehealth or in-person (began therapy in-person and switched to telehealth prior to week 4); thus, our final sample comprised 53 youth and their parents. Families were randomized into FBT alone, FBT with parent cognitive remediation therapy (CRT) or FBT with adolescent CRT. Families completed CRT and FBT over a 2-h block, 1-h CRT followed by 1-h FBT. Inclusion criteria were age 12–18 years, AN diagnosis, weight less than 95% median BMI, medically stable for outpatient care and with both biological parents willing to participate. Exclusion criteria included history of brain injury or diagnosis potentially impacting executive functioning for either parents or youth or current use of atypical antipsychotic medication. All procedures were approved by the institutional review board and both parents and adolescents provided informed consent/assent.

At baseline, adolescents were aged 15.45 years (*SD* = 1.59), with 45 assigned female and 8 assigned male at birth. The sample was overwhelmingly White (94%, *n* = 50) and non-Hispanic (98%, *n* = 53). Average BMI *z*-score at baseline was −0.18 (*SD* = 0.74), with 43% of the sample meeting criteria for severe malnutrition. Average duration of illness was 13.51 months at data collection (*SD* = 15.18).

### 2.2 ∣ Measures

#### 2.2.1 ∣ Working Alliance Inventory–Short-Revised (WAI-SR; Hatcher and Gillaspy 2006)

The WAI-SR assesses patient perceptions of therapeutic alliance during therapy. The reliability and validity of the WAI-SR in an outpatient setting are well established (Munder et al. 2010). The WAI-SR is a 12-item measure with a 5-item Likert-type response scale. Participants are asked to rate how often they experience specific components of a strong working alliance with their therapist, resulting in a total score as well as three subscales: goals, task and bond. Goals encompass overall what would be gained from treatment, task entails what needs to be done to reach goals and bond relates to trust and confidence in the therapist (Bordin 1979). Internal consistency in our sample was good to excellent α = 0.88–0.95.

#### 2.2.2 ∣ Working Alliance Inventory–Self-Report Therapist (WAI-SRT; Hatcher and Gillaspy 2006)

Therapists also completed the WAI-SRT. This questionnaire assesses the same three subscales, bond, task and goals, and is a 10-item variation of the survey with a 5-item Likert-type response scale. Scores on the WAI-SRT have demonstrated acceptable construct validity and, in our sample, good internal consistency α = 0.88.

### 2.3 ∣ Procedures

Adolescents, parents and therapists completed the WAI at 4 weeks after baseline (T2). All families received 15 sessions of FBT over 6 months, with an additional 15 sessions of CRT for participants randomized to adolescent (*n* = 19) or parent CRT (*n* = 20). In-person treatment sessions were halted in March 2020 due to COVID-19–related shutdowns. There were 20 families who completed their first 4 weeks in-person and 33 families who completed at least their first four treatment sessions via a secure web-based video conferencing platform. We had four therapists in the study, two of whom saw participants both prior to and after the shift to telehealth, one who only saw participants who began in-person and a fourth who only saw participants via telehealth.

### 2.4 ∣ Statistical Analyses

As there was no difference in patient (adolescent and parent) ratings of therapeutic alliance across all therapists, data were collapsed across therapists for all analyses. For participant perception of alliance, we explored potential covariates using correlation analysis or *t*-tests. Due to heterogeneity of variance, we were not able to conduct a 2 (treatment modality) × 3 (family member) analysis of (co)variance. Thus, we examined differences in therapeutic alliance in-person (*n* = 20) to telehealth (*n* = 33) by family member. As there are no clinical cut-offs for the WAI, we examined whether early treatment alliance differed across participants who completed treatment (*n* = 46) compared with those who dropped out of treatment (*n* = 8). To examine therapist perception of alliance by treatment modality, we included only the two therapists who provided treatment that began both pre and post the pandemic-related shutdown (patient *n* = 20). When a covariate was needed and assumptions were met, we used ANCOVA; otherwise, we used independent samples *t*-tests and Mann–Whitney *U* test when variables did not meet parametric assumptions. For participants with missing data, we used mean replacement to maximize our sample.

## 3 ∣ Results

### 3.1 ∣ Sociodemographic and Clinical Associations

Adolescents who began with in-person treatment did not differ from those who received telehealth treatment by sex, age or body mass index at presentation (small effect sizes, *p*s > 0.05). Older age in adolescents was associated with higher self-reported bond with therapist (*r* = 0.43, *p* < 0.01), but no other self or therapist-report WAI subscales (*r*s = −0.11 to 0.23, *p*s > 0.05). BMI *z*-score was not associated with adolescent or therapist report of alliance (*r*s = −0.23 to 0.27, *p*s > 0.05). Girls reported higher goal-related, *t*(43) = −2.10, *p* = 0.04, Hedge's *g* = −0.90, and task-related alliance, *t*(43) = −2.27, *p* = 0.03, Hedge's *g* = −0.98, with their therapist, but there were no other differences in self or therapist-reported alliance across sex (*z*s = −1.27 to 0.77, *p*s > 0.05). We found no significant differences in alliance across treatment condition (*z*s = 1.11–1.90, *η*^2^ = 0.04–0.11, *p*s > 0.05). Where alliance significantly differed across clinical or sociodemographic variables (e.g., age in adolescent therapeutic bond), this was controlled for in subsequent analyses. Means and standard deviations for working alliance are shown in Table 1.

### 3.2 ∣ Working Alliance

#### 3.2.1 ∣ Working Alliance and Dropout

Per adolescent report (*n* = 45), we saw no significant difference across treatment completers and those who later dropped out of treatment in task-related alliance, *U* = 158.00, *p* = 0.050. However, we saw significantly higher therapeutic bond, *t*(44) = −3.75, *p* < 0.001, Hedge's *g* = −1.75, and goal-related alliance, *t*(44) = −2.46, *p* = 0.02, Hedge's *g* = −1.15, for treatment completers. For paternal report, there were no significant differences across task-related alliance, *t*(48) = −0.09, *p* = 0.93, Hedges' *g* = −0.04, therapeutic bond, *t*(48) = −1.08, *p* = 0.29, Hedge's *g* = −0.43, or goal-related alliance, *t*(6.71) = −0.87, *p* = 0.42, Glass' delta = −0.51, between treatment completers and noncompleters. We did not see significant differences in maternal-report task-related alliance, *t*(48) = −0.56, *p* = 0.58, Hedge's *g* = −0.26, or goal-related alliance, *t*(48) = −0.08, *p* = 0.94, Hedge's *g* = −0.04, although we did see significantly higher therapeutic bond, *t*(−1.24) = −2.64, *p* = 0.02, Glass' delta = −0.58, in treatment completers compared noncompleters. Finally, therapist-reported task-related alliance did not differ across treatment completers and noncompleters, *t*(6.42) = −2.13, *p* = 0.07, Glass' delta = −1.88. We did see significantly higher therapist-related therapeutic bond (*U* = 222.5, *p* < 0.01) and goal-related alliance (*U* = 218.5, *p* = 0.01) in treatment completers compared to those who dropped out.

#### 3.2.2 ∣ Adolescent Working Alliance

Results revealed no significant difference in task-related alliance across telehealth and in-person modalities controlling for patient sex, *F*(2, 42) = 3.00, *p* = 0.06, partial *η*^2^ = 0.13, despite a medium effect. Adolescents (*n* = 45) reported significantly higher self-reported bond across modality, controlling for age, *F*(2, 42) = 6.54, *p* < 0.01, partial *η*^2^ = 0.24, with greater bond for in-person sessions compared to telehealth. We also found significantly greater goal-related alliance for in-person sessions compared to telehealth, controlling for sex, with a large effect *F*(2, 42) = 3.54, *p* = 0.04, partial *η*^2^ = 0.14.

#### 3.2.3 ∣ Paternal Working Alliance

Similarly, we did not detect differences for fathers (*n* = 49) on task-related alliance, *t*(47) = 1.56, *p* = 0.13, Hedge's *g* = 0.45, or bond, *t*(47) = 1.34, *p* = 0.19, Hedge's *g* = 0.38, or goal-related alliance, *t*(48) = 1.68, *p* = 0.10, Hedge's *g* = 0.48, between telehealth and in-person attendees.

#### 3.2.4 ∣ Maternal Working Alliance

Maternal participants (*n* = 49) reported greater task-related alliance, *t*(47) = 3.49, *p* < 0.01, Hedge's *g* = 1.00, bond, *t*(47) = 2.44, *p* = 0.02, Hedge's *g* = 0.70, and goal-related alliance, *t*(47) = 3.54, *p* < 0.001, Hedge's *g* = 1.01, during in-person sessions compared with telehealth.

#### 3.2.5 ∣ Therapist Working Alliance

Finally, we saw no significant differences across telehealth and in-person sessions for therapist-reported (*n* = 20) task-related alliance (*U* = 32.00, *p* = 0.24), therapeutic bond (*U* = 45.00, *p* = 0.85) or goal-related alliance, *t*(18) = 1.37, *p* = 0.21, Glass' delta = 0.51.

## 4 ∣ Discussion

This study was a cross-sectional post hoc analysis of differences in therapeutic alliance for families and therapists in FBT for AN who were enrolled in a clinical trial over the pandemic-related transition to telehealth. Overall, our results were mixed. For therapists and fathers, we found no significant differences between telehealth and in-person sessions. In both adolescents and mothers, we saw significantly higher therapeutic bond and goal-related alliance at 4 weeks for in-person sessions, with mothers also reporting significantly higher task-related alliance in-person. Due to the relatively small size of our sample, results should be interpreted with caution. However, they do emphasize concerns of some clinicians that both youth and caregivers may have difficulties in building a strong therapeutic alliance via virtual platforms (Matheson, Bohon, and Lock 2020).

Findings of lower therapeutic bond, goal and task-related alliance for telehealth sessions may be consistent with parent feedback that it can be difficult building therapeutic connections via virtual platform (Couturier et al. 2022). This is consistent with qualitative data from Lewis et al. (2021), who found that 40% of participants stated that they felt the transition to telehealth affected the quality or effectiveness of care they received. Importantly, the families receiving treatment via telehealth were receiving it during the COVID-19 pandemic-related shutdown. We cannot rule out the possibility that the impact of isolation during quarantine could have reduced alliance due to ‘Zoom fatigue’ that many experienced in the first months of the pandemic. Future studies may wish to assess whether adaptations to telehealth FBT mitigate these effects—for example, using in-person treatment for early sessions prior to shifting online, randomizing participants to telehealth and remote groups or seeing whether patient *preference* for online/in-person is important for therapeutic alliance (Couturier et al. 2022). Although data on youth-reported alliance is mixed, maternal-rated alliance may be particularly important for weight gain in FBT (Graves et al. 2017). Indeed, in our sample, lower early working alliance predicted future treatment dropout, highlighting the need to better understand how patients experience the transition to telehealth. Contrary to possible concerns by therapists that establishing strong alliance is more difficult via telehealth, therapists in the original study who saw patients in both modalities did not report weaker therapeutic alliance for telehealth.

Strengths of this study include the use of a well-validated measure of working alliance, reports from both mothers and fathers and that all families participating in treatment contributed data (minimizing self-selection bias). Limitations include the relatively small sample size and low diversity in our sample, limiting our capacity to generalize results. In addition, WAI scores are sums of all items, creating a continuous variable from ordinal responses. While higher scores indicate better alliance, it is unclear what would be a clinically significant difference in scores. Overall, findings for the use of FBT for youth with AN via telehealth were mixed. Future studies should further examine findings in mothers and youth to determine whether these impact youth and family outcomes.

## Figures and Tables

**TABLE 1 ∣:** Means and standard deviations for working alliance across reporter and telehealth versus in-person.

	Adolescent		Father		Mother		Therapist	
	In-person (*n* = 18)	Telehealth (*n* = 27)	In-person (*n* = 20)	Telehealth (*n* = 29)	In-person (*n* = 20)	Telehealth (*n* = 29)	In-person (*n* = 20)	Telehealth (*n* = 26)
Task-related alliance	11.7 (4.6)	10.2 (5.2)	14.1 (2.9)	12.5 (4.0)	15.7** (3.3)	12.2** (3.5)	12.0 (2.1)	10.8 (2.4)
Therapeutic bond	15.8** (4.5)	14.3** (4.1)	15.8 (3.1)	15.3 (3.5)	17.3* (3.1)	15.1* (3.1)	18.9 (1.8)	19.4 (1.3)
Goal-related alliance	14.4* (4.4)	11.81* (5.9)	16.2 (3.2)	14.9 (3.2)	17.0** (2.4)	13.7** (3.7)	12.7 (1.6)	11.1 (2.4)

*Note:* The number of items on the WAI-SR form differs across client self-report and therapist report; thus, scores cannot be directly compared from patient to therapist.

* *p* <0.05, and

** *p* < 0.01.

## Data Availability

Data from this project are available from the corresponding author upon reasonable request.

## Summary

- Therapists may need to focus on alliance building with youth with AN and their mothers in telehealth FBT.
- Modifications to FBT may need to be made to promote greater therapeutic alliance.
- Future research should explore the impact of alliance by treatment modality on clinical outcomes.

## References

[R1] Andrews E, Berghofer K, Long J, Prescott A, and Caboral-Stevens M. 2020. “Satisfaction With the Use of Telehealth During COVID-19: An Integrative Review.” International Journal of Nursing Studies Advances 2: 100008. 10.1016/j.ijnsa.2020.100008. 33083791 PMC7564757

[R2] Bordin ES 1979. “The Generalizability of the Psychoanalytic Concept of the Working Alliance.” Psychotherapy: Theory, Research & Practice 16, no. 3: 252–260. 10.1037/h0085885.

[R3] Couturier J, Pellegrini D, Grennan L, 2022. “A Qualitative Evaluation of Team and Family Perceptions of Family-Based Treatment Delivered by Videoconferencing (FBT-V) for Adolescent Anorexia Nervosa During the COVID-19 Pandemic.” Journal of Eating Disorders 10, no. 1: 111. 10.1186/s40337-022-00631-9. 35883167 PMC9321306

[R4] Cowan KE, McKean AJ, Gentry MT, and Hilty DM. 2019. “Barriers to Use of Telepsychiatry: Clinicians as Gatekeepers.” Mayo Clinic Proceedings 94, no. 12: 2510–2523. 10.1016/j.mayocp.2019.04.018. 31806104

[R5] Fisk M, Livingstone A, and Pit SW. 2020. “Telehealth in the Context of COVID-19: Changing Perspectives in Australia, the United Kingdom, and the United States.” Journal of Medical Internet Research 22, no. 6: e19264. 32463377 10.2196/19264 PMC7286230

[R6] Forsberg S, LoTempio E, Bryson S, Fitzpatrick KK, Le Grange D, and Lock J. 2014. “Parent–Therapist Alliance in Family-Based Treatment for Adolescents With Anorexia Nervosa.” European Eating Disorders Review 22, no. 1: 53–58. 10.1002/erv.2242. 23861093

[R7] Frayn M, Fojtu C, and Juarascio A. 2021. “COVID-19 and Binge Eating: Patient Perceptions of Eating Disorder Symptoms, Tele-Therapy, and Treatment Implications.” Current Psychology 40, no. 12: 6249–6258. 10.1007/s12144-021-01494-0. 33623352 PMC7891466

[R8] Graves TA, Tabri N, Thompson-Brenner H, 2017. “A Meta-Analysis of the Relation Between Therapeutic Alliance and Treatment Outcome in Eating Disorders.” International Journal of Eating Disorders 50, no. 4: 323–340. 10.1002/eat.22672. 28152196

[R9] Hatcher RL, and Gillaspy JA. 2006. “Development and Validation of a Revised Short Version of the Working Alliance Inventory.” Psychotherapy Research 16, no. 1: 12–25. 10.1080/10503300500352500.

[R10] Horvath AO, Del Re AC, Flückiger C, and Symonds D. 2011. “Alliance in Individual Psychotherapy.” Psychotherapy 48, no. 1: 9–16. 10.1037/a0022186. 21401269

[R11] Lewis YD, Elran-Barak R, Grundman-Shem Tov R, and Zubery E. 2021. “The Abrupt Transition From Face-to-Face to Online Treatment for Eating Disorders: A Pilot Examination of Patients' Perspectives During the COVID-19 Lockdown.” Journal of Eating Disorders 9, no. 1: 31. 10.1186/s40337-021-00383-y. 33673876 PMC7934980

[R12] Linardon J, Messer M, Rodgers RF, and Fuller-Tyszkiewicz M. 2022. “A Systematic Scoping Review of Research on COVID-19 Impacts on Eating Disorders: A Critical Appraisal of the Evidence and Recommendations for the Field.” International Journal of Eating Disorders 55, no. 1: 3–38. 10.1002/eat.23640. 34773665 PMC8646470

[R13] Madigan S, Racine N, Cooke JE, and Korczak DJ. 2021. “COVID-19 and Telemental Health: Benefits, Challenges, and Future Directions.” Canadian Psychology/Psychologie Canadienne 62, no. 1: 5–11. 10.1037/cap0000259.

[R14] Martin-Wagar CA, Holmes S, and Bhatnagar KAC. 2019. “Predictors of Weight Restoration in a Day-Treatment Program That Supports Family-Based Treatment for Adolescents With Anorexia Nervosa.” Eating Disorders 27, no. 4: 400–417. 10.1080/10640266.2018.1528085. 30358497

[R15] Matheson BE, Bohon C, and Lock J. 2020. “Family-Based Treatment via Videoconference: Clinical Recommendations for Treatment Providers During COVID-19 and Beyond.” International Journal of Eating Disorders 53, no. 7: 1142–1154. 10.1002/eat.23326. 32533799 PMC7323318

[R16] Munder T, Wilmers F, Leonhart R, Linster HW, and Barth J. 2010. “Working Alliance Inventory-Short Revised (WAI-SR): Psychometric Properties in Outpatients and Inpatients.” Clinical Psychology & Psychotherapy 17, no. 3: 231–239. 10.1002/cpp.658. 20013760

[R17] Pereira T, Lock J, and Oggins J. 2006. “Role of Therapeutic Alliance in Family Therapy for Adolescent Anorexia Nervosa.” International Journal of Eating Disorders 39, no. 8: 677–684. 10.1002/eat.20303. 16937386

[R18] Raykos BC, Erceg-Hurn DM, Hill J, Campbell BNC, and McEvoy PM. 2021. “Positive Outcomes From Integrating Telehealth Into Routine Clinical Practice for Eating Disorders During COVID-19.” International Journal of Eating Disorders 54, no. 9: 1689–1695. 10.1002/eat.23574. 34184797

[R19] Shirk SR, Karver MS, and Brown R. 2011. “The Alliance in Child and Adolescent Psychotherapy.” Psychotherapy 48, no. 1: 17–24. 10.1037/a0022181. 21401270

[R20] Timko CA, Bhattacharya A, Fitzpatrick KK, 2021. “The Shifting Perspectives Study Protocol: Cognitive Remediation Therapy as an Adjunctive Treatment to Family Based Treatment for Adolescents With Anorexia Nervosa.” Contemporary Clinical Trials 103: 106313. 10.1016/j.cct.2021.106313. 33539993 PMC8489286

